# Supplementary material for: GPR174 knockdown enhances blood flow recovery in hindlimb ischemia mice model by upregulating AREG expression
Source: Nat Commun. 2022 Dec 6;13:7519. doi: 10.1038/s41467-022-35159-8 (PMC9727025; doi:10.1038/s41467-022-35159-8)

GPR174 Knockdown Enhances Blood Flow Recovery in Hindlimb Ischemia Mice Model by Upregulating AREG Expressions

Supplementary Figures

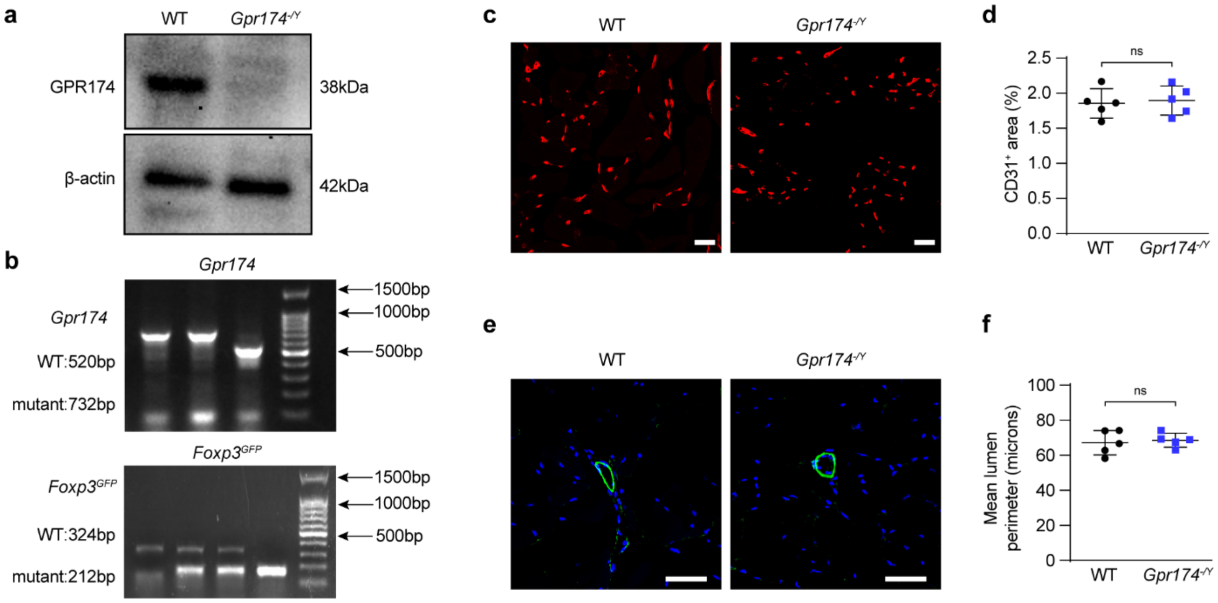

Supplementary Fig. 1. Global GPR174 deficiency has no effects on basal vascularization. **a** Immunoblot analysis of GPR174 expression in gastrocnemius from C57BL/6J and global GPR174-deficient mice. Data are obtained from three independent experiments and representative images are shown. **b** Representative PCR analysis on genomic DNA of wild-type, *Gpr174*$^{-/Y}$ mice, and *Foxp3*^{GFP} mice. **c**, **d** Representative immunofluorescent images of CD31 staining in gastrocnemius cross sections and quantification of CD31 in WT and *Gpr174*$^{-/Y}$ mice at baseline (n=5). Scale bar, 50 μm. **e**, **f** Representative images of αSMA (green) and DAPI (blue) immunostainings in gastrocnemius cross sections and quantification of lumen perimeter in wild-type and *Gpr174*$^{-/Y}$ mice (n=5; wild-type: 38 arteries from 5 mice, *Gpr174*$^{-/Y}$: 37 arteries from 5 mice). Scale bar, 50 μm. For all statistical plots, the data are presented as mean ± SD. Two-tailed unpaired t-tests in (**d**, **f**). Source data are provided as a Source Data file.

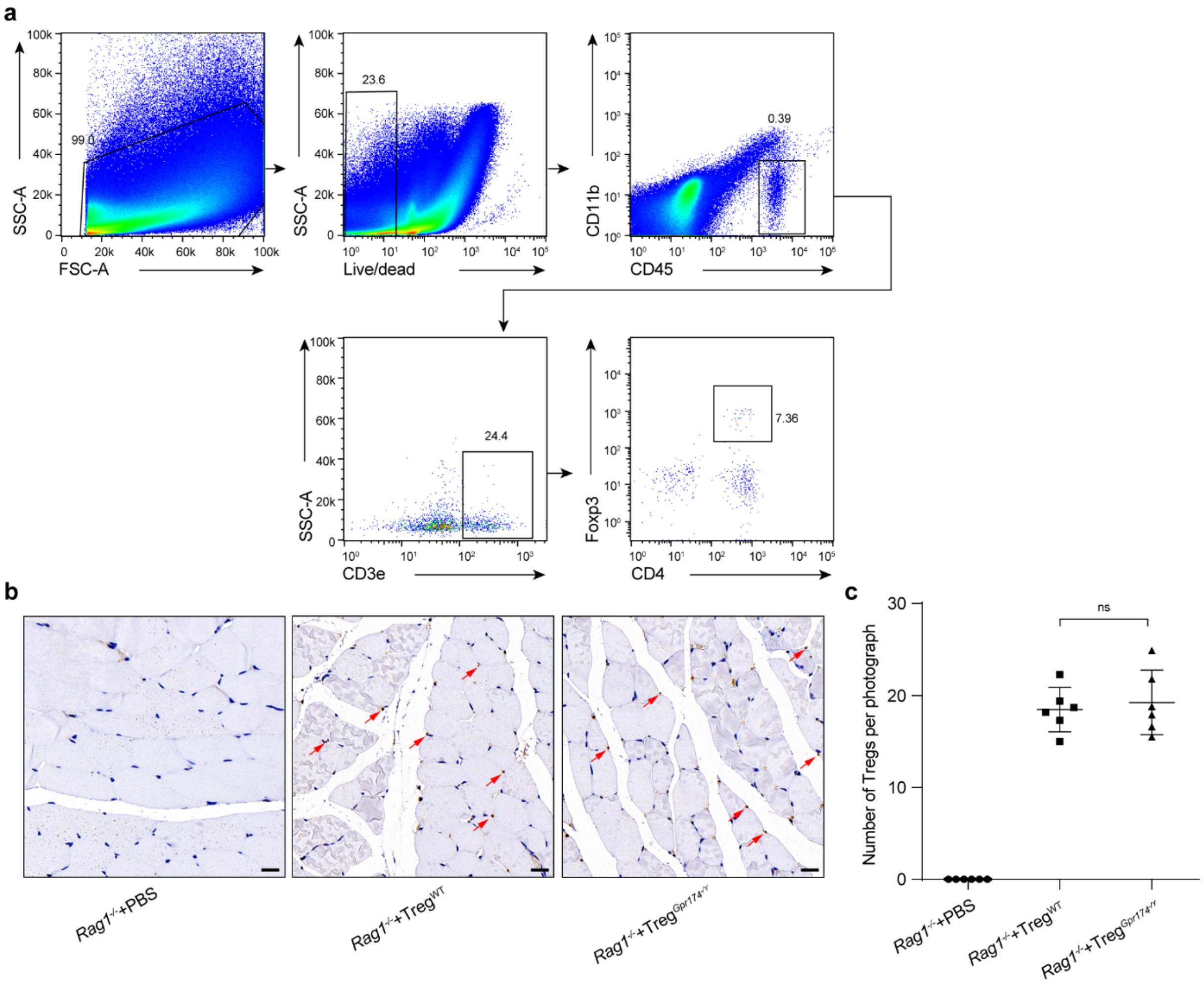

Supplementary Fig. 2. Tregs were injected into *Rag1$^{-/-}$* mice calf muscles. a Gating strategy for sorting CD4$^+$Foxp3^{GFP} Tregs using flow cytometry in WT and *Gpr174$^{-/Y}$* mice. **b** Representative immunofluorescent images of Foxp3 staining in ischemic gastrocnemius from *Rag1$^{-/-}$* mice injected with PBS or Tregs. Scale bar, 50 µm. **c** Quantification of Foxp3$^+$ Tregs in ischemic gastrocnemius cross sections of *Rag1$^{-/-}$* mice receiving Tregs (n=6). For all statistical plots, the data are presented as mean ± SD. One-way ANOVA with Bonferroni multiple comparisons test in (**c**). Source data are provided as a Source Data file.

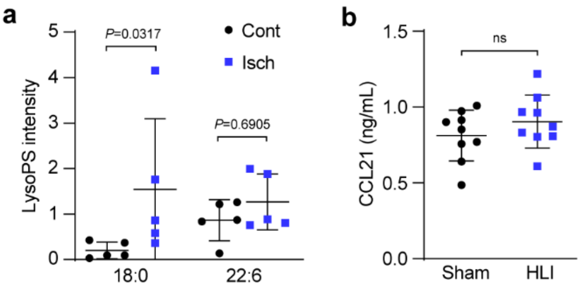

Supplementary Fig. 3. Lysophosphatidylserine (LysoPS) promotes GPR174-Gαs signaling. **a** LysoPS abundance in the muscle tissues from wild-type mice 5 days after HLI (n=5). **b** Quantification of CCL21 in serum collected from wild-type mice 5 days after HLI (n=9). For all statistical plots, the data are presented as mean ± SD. Two-tailed unpaired t-tests in (**a**, **b**). Source data are provided as a Source Data file.

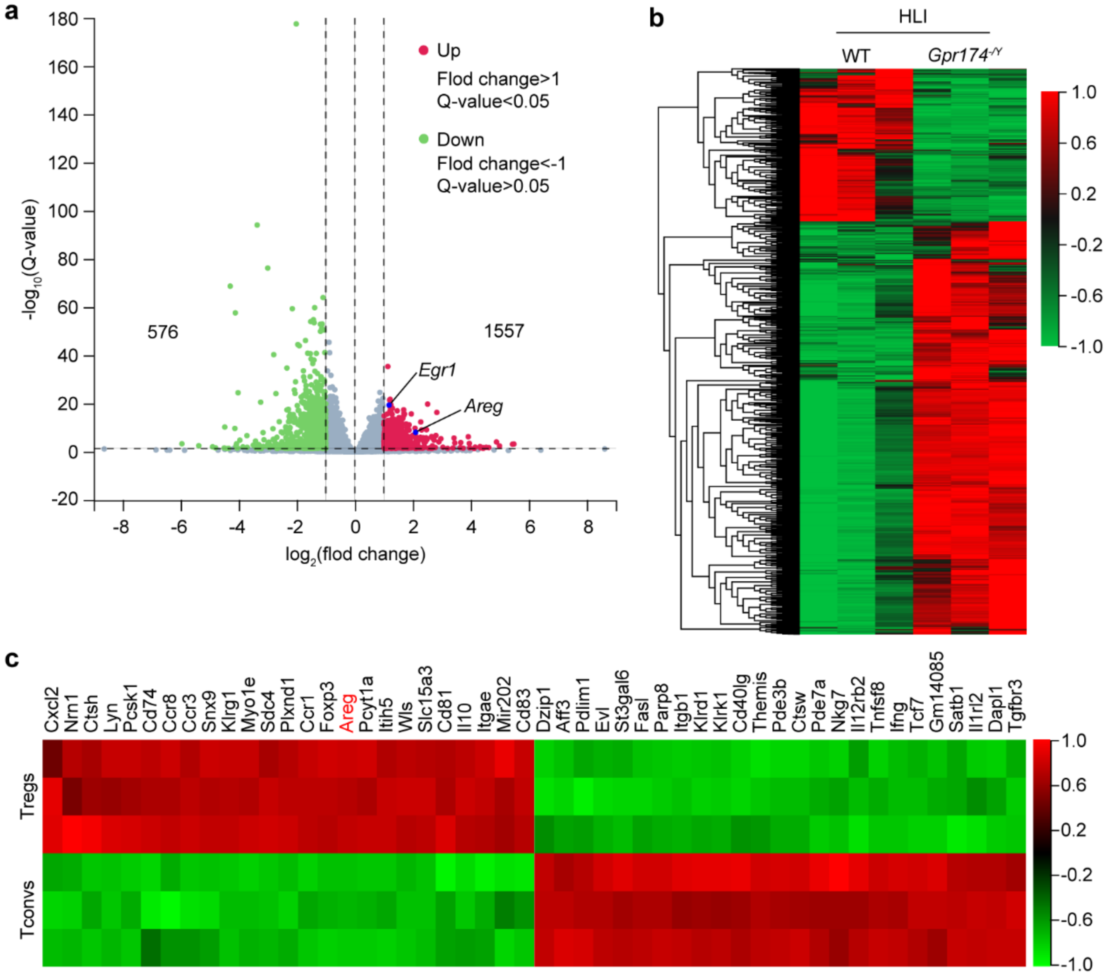

Supplementary Fig. 4. RNA-seq analysis of gene expression profile in muscles from WT controls and *GPR174*$^{-/Y}$ mice post HLI. a Volcano plot of differentially expressed genes in muscles of GPR174KO compared with wild-type 7 days post HLI (n=3). Red dots represent up-regulated DEGs in ischemic muscles from GPR174KO versus WT controls whereas green dots indicate down-regulated DEGs. **b** Heat map of differentially expressed genes in muscles of GPR174KO compared with wild-type 7 days post HLI. **c** Heatmap of genes significantly up- or down-regulated in Tregs from injured muscle compared with Tregs from spleen by analyzing the microarray datasets GSE50096. Genes with absolute fold change>2 and adjusted q-value<0.05 are defined as differentially expressed genes (DEGs).

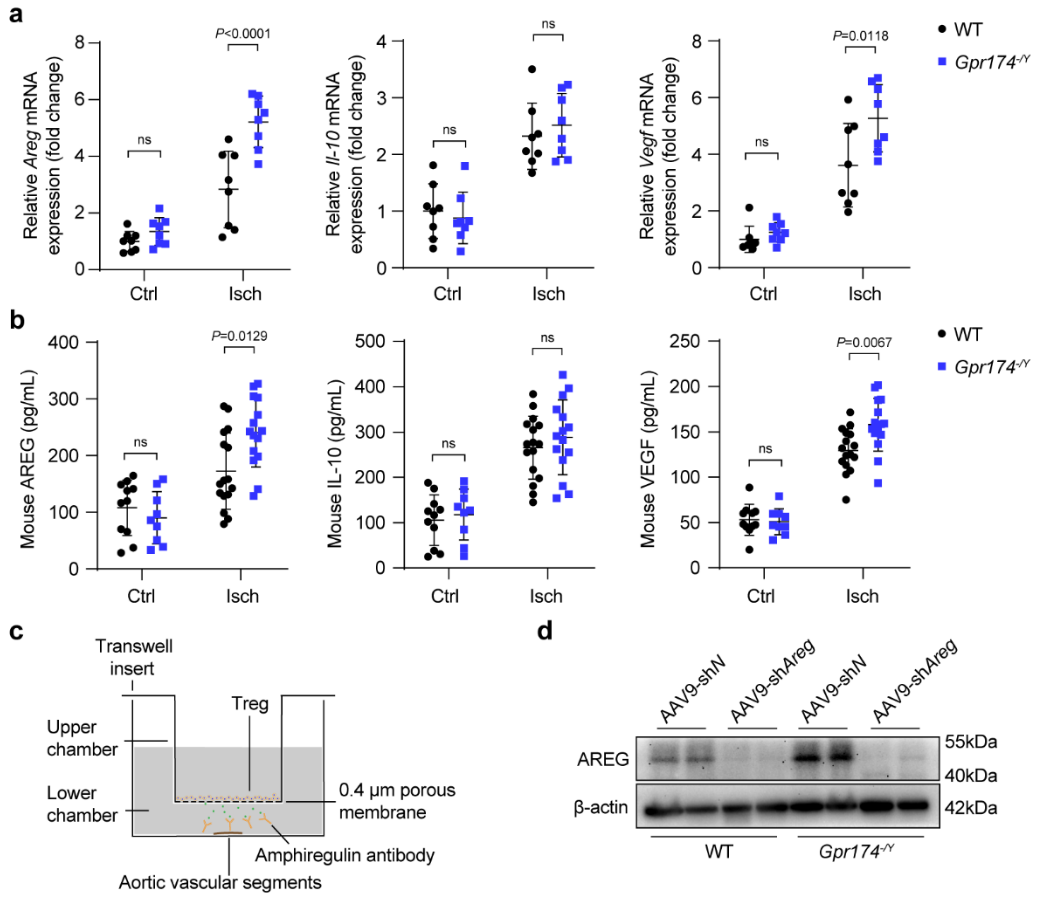

Supplementary Fig. 5. AREG expression increased in gastrocnemius after HLI. a Relative mRNA levels of *Areg*, *Il-10*, *Vegf* in gastrocnemius from wild-type and *Gpr174*$^{-/Y}$ mice 7 days post HLI (n=8). **b** Serum AREG, IL-10, and VEGF protein content in wild-type and *Gpr174*$^{-/Y}$ mice 7 days post HLI (n=10 for nonischemic WT mice; n=9 for nonischemic *Gpr174*$^{-/Y}$ mice; n=16 for ischemic WT mice; n=15 for ischemic *Gpr174*$^{-/Y}$ mice). **c** Scheme of thoracic aorta ring sprouting assay. **d** Western blotting analysis of AREG levels in ischemic muscle from WT controls and *Gpr174*$^{-/Y}$ mice injected with AAV9-sh*Areg*. Data are obtained from four independent experiments and representative images are shown. For all statistical plots, the data are presented as mean ± SD. Two-way ANOVA with Bonferroni multiple comparisons test in (**a**, **b**). Source data are provided as a Source Data file.

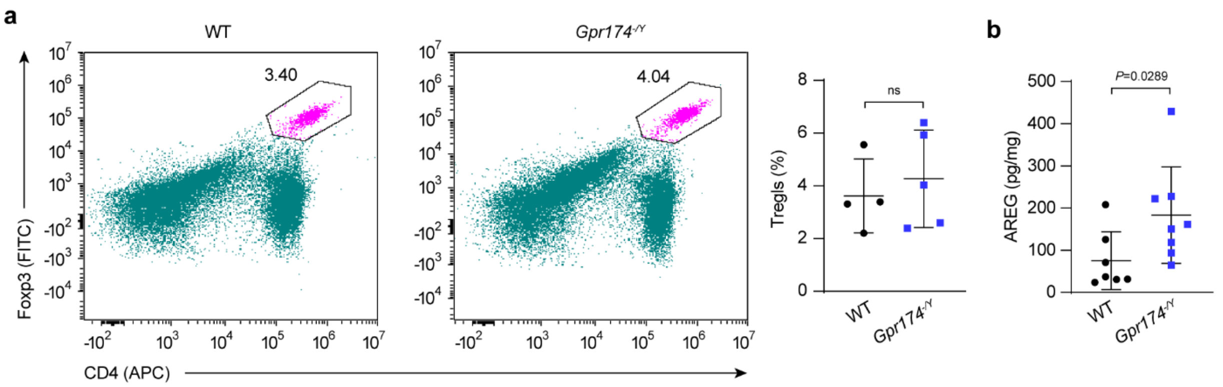

Supplementary Fig. 6. Tregs infiltrated in Matrigel plugs secrete AREG. a Representative flow cytometric dot plots of Tregs in Matrigel plugs (n=4 for WT; n=5 for *Gpr174*$^{-/Y}$). **b** AREG levels in Matrigel plugs (n=7 for WT; n=8 for *Gpr174*$^{-/Y}$). For all statistical plots, the data are presented as mean ± SD. Two-tailed unpaired t-tests in (**a**, **b**). Source data are provided as a Source Data file.

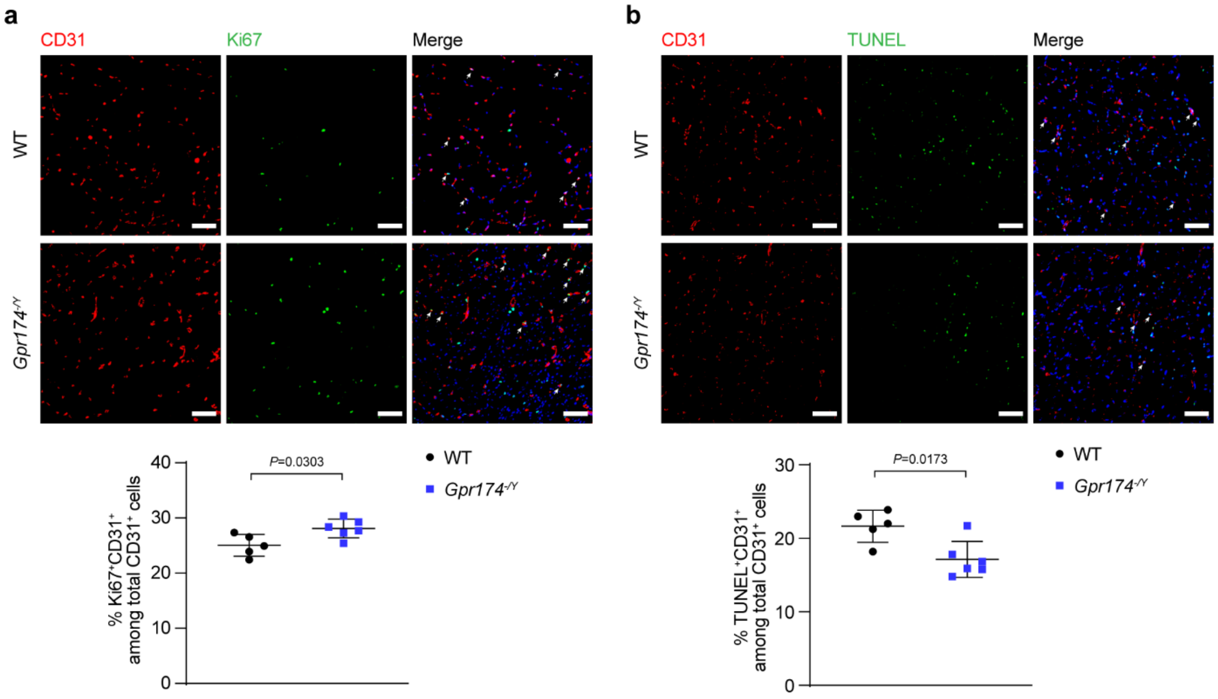

Supplementary Fig. 7. Proliferation and apoptosis of Endothelial cells 3 days after HLI. a Representative immunofluorescent image of CD31 (red), Ki67 (green) and DAPI (blue) and quantification of CD31$^+$Ki67$^+$ cells in muscle cross sections 3 days after HLI (n=5 for WT; n=6 for *Gpr174*$^{-/Y}$). Scale bar, 50 µm. **b** Representative immunofluorescent images of CD31 (red), TUNEL (green), and DAPI (blue) staining and quantification of CD31$^+$TUNEL$^+$ cells in muscle cross sections 3 days after HLI (n=5 for WT; n=6 for *Gpr174*$^{-/Y}$). Scale bar, 50 µm. For all statistical plots, the data are presented as mean ± SD. Two-tailed unpaired t-tests in (**a**, **b**). Source data are provided as a Source Data file.

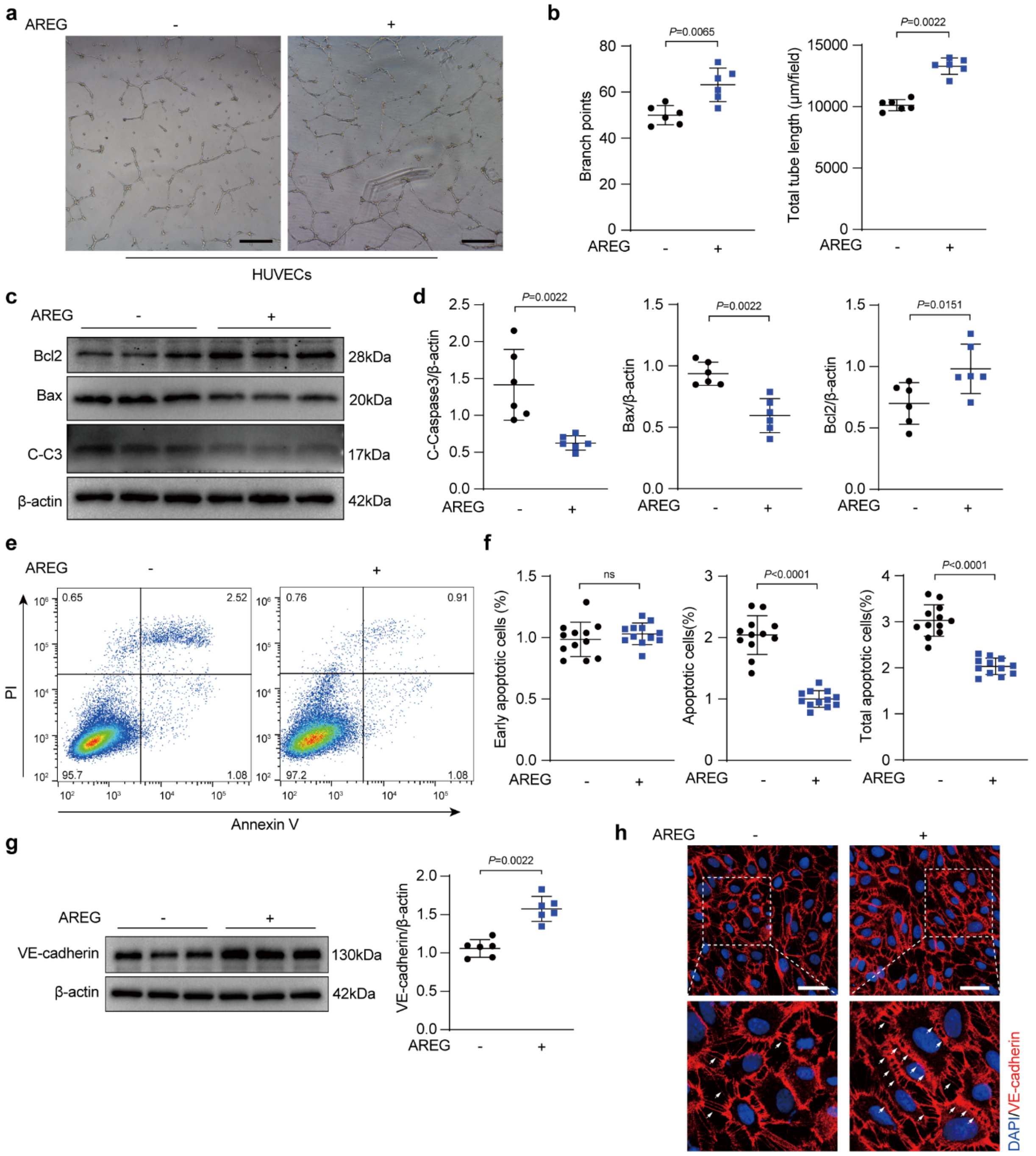

Supplementary Fig. 8. AREG mitigates apoptosis and promotes adherens junctions in endothelial cells. a, **b** Representative images of capillary-like structures and quantification of branch points and total tube length in HUVECs stimulated with recombinant AREG for 16h (n=6). Scale bar, 200 μm. **c**, **d** Western blotting analysis of apoptosis and anti-apoptosis associated genes in endothelial cells stimulated with recombinant AREG for 24 hours (n=6). **e**, **f** Flow cytometric analysis of apoptotic cells in endothelial cells treated with recombinant

AREG for 24 hours (n=12). **g** Western blotting analysis of VE-cadherin levels in endothelial cells (n=6). **h** Representative immunofluorescent images of VE-cadherin (Red) and DAPI (Blue) immunostainings in endothelial cells. Scale bar, 50 μm. For all statistical plots, the data are presented as mean ± SD. Two-tailed unpaired t-tests in (**b**, **d**, **f**, **g**). Source data are provided as a Source Data file.

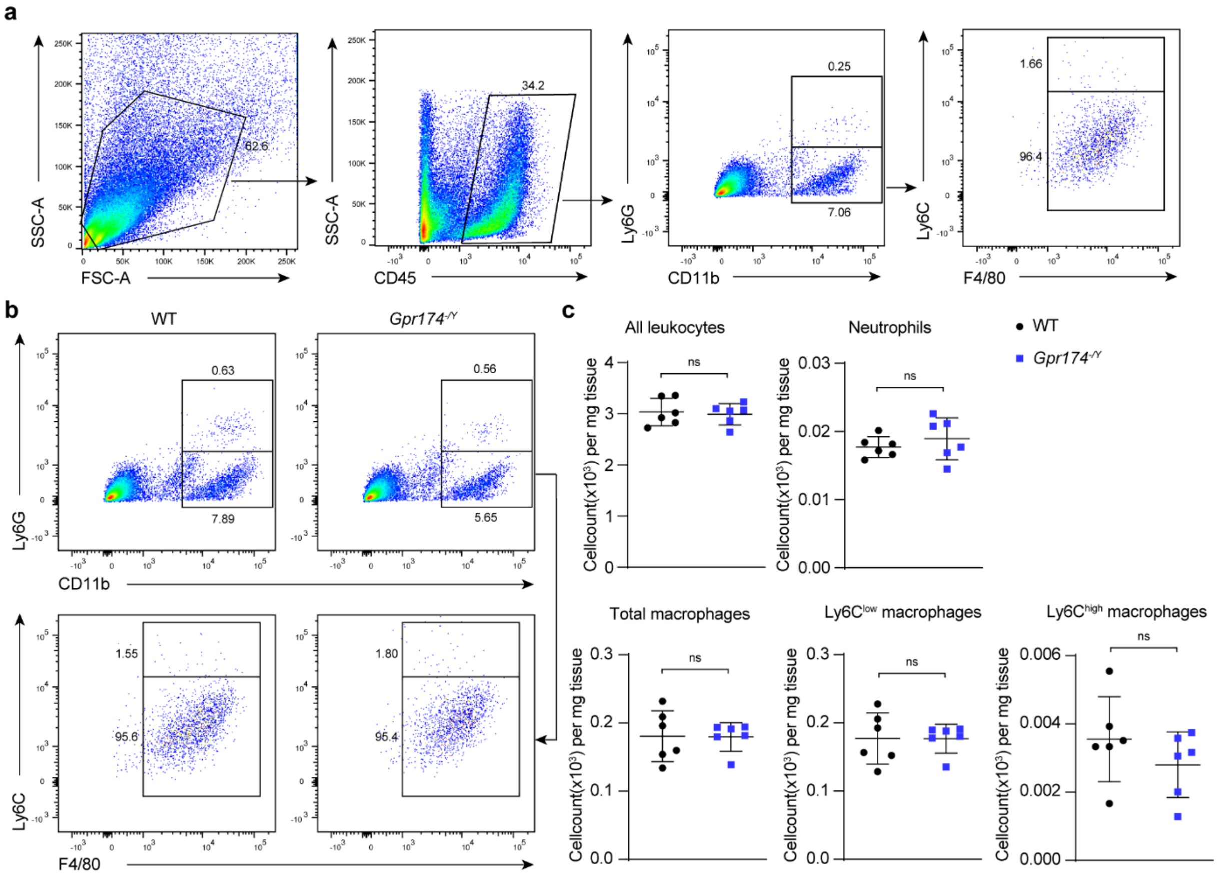

Supplementary Fig. 9. Global GPR174 deficiency has no effects on inflammation at baseline. **a** Flow cytometry gating strategy for leukocytes of gastrocnemius from WT and *Gpr174*$^{-/Y}$ mice. **b**, **c** Representative flow cytometric dot plots to determine neutrophils, macrophages in the non-ligated gastrocnemius of WT and *Gpr174*$^{-/Y}$ mice (n=6). For all

statistical plots, the data are presented as mean ± SD. Two-tailed unpaired t-tests in (**c**). Source data are provided as a Source Data file.

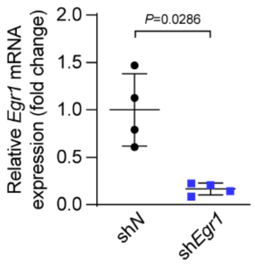

Supplementary Fig. 10. Knockdown efficiency of *Egr1*. Quantitative polymerase chain reaction performed to verify lentivirus-mediated *Egr1* knockdown in Tregs (n=4). The data are presented as mean ± SD. Two-tailed unpaired t-test. Source data are provided as a Source Data file.

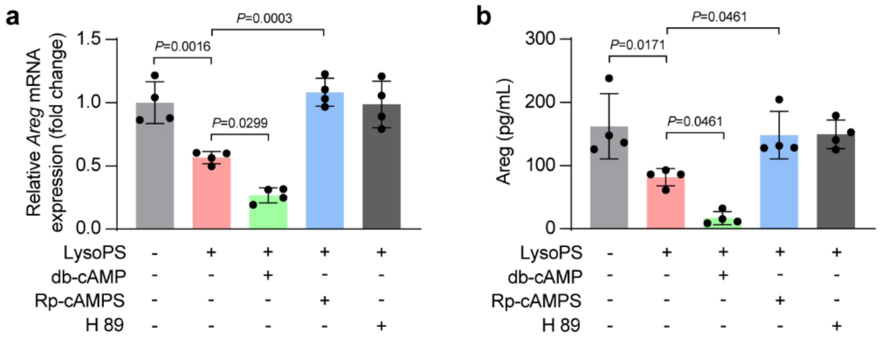

Supplementary Fig. 11. LysoPS inhibits AREG expression in Tregs through cAMP/PKA pathway. a, b qRT-PCR (a) and ELISA (b) analysis of AREG expression in Tregs receiving different treatments for 24 h, n=4 biologically independent samples. For all statistical plots, the data are presented as mean ± SD. Two-way ANOVA with Bonferroni multiple comparisons test in (**a**, **b**). Source data are provided as a Source Data file.

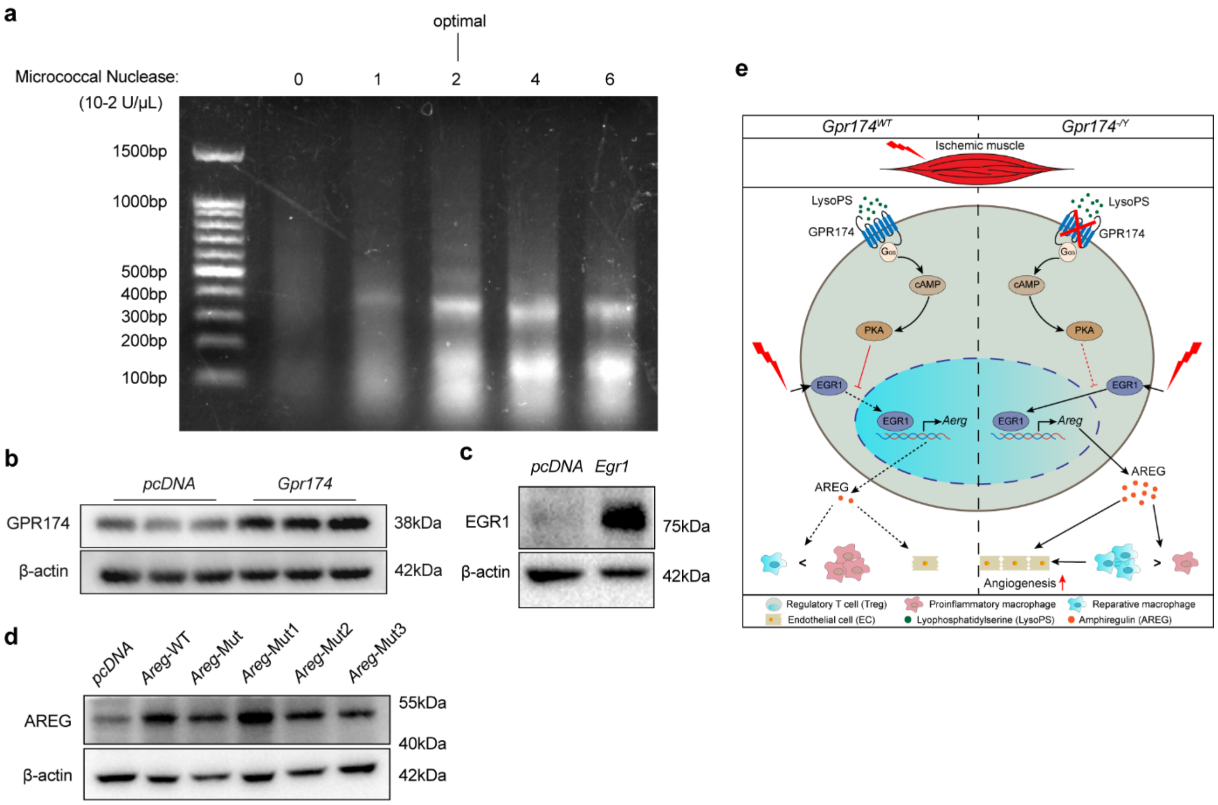

Supplementary Fig. 12. Preparation of chromatin immunoprecipitation and dual luciferase assay. a Optimization of chromatin fragmentation. The concentration of micrococcal nuclease for chromatin fragmentation in ChIP assay was determined by examining different of concentration and the one that generated DNA binds were between 200 and 1,000bp with a more intense ladder of bands occurring at approximately 160, 320, and 480 bp was considered optimal. Data are obtained from three independent experiments and representative images are shown. **b** Western blotting performed to verify GPR174 overexpression in HEK293A cells. Data are obtained from three independent experiments. **c** Immunoblot analysis of EGR1 overexpression in HEK293A cells. Data are obtained from three independent experiments and representative images are shown. **d** Immunoblot analysis of AREG (WT and Mutants) overexpression in HEK293A cells. Data are obtained from three independent experiments and representative images are shown. **e** A schematic model of GPR174-mediated signaling in Tregs

post HLI. LysoPS increases GPR174 activity and then activates Gαs/cAMP/PKA signaling pathway, thereby inhibiting EGR1 nuclear accumulation in Treg. EGR1 elevates AREG expression by binding to the promoter *Areg*. AREG increases endothelial cell proliferation and ameliorates inflammatory response and endothelial cell apoptosis, which improves angiogenesis in ischemic muscle tissues.

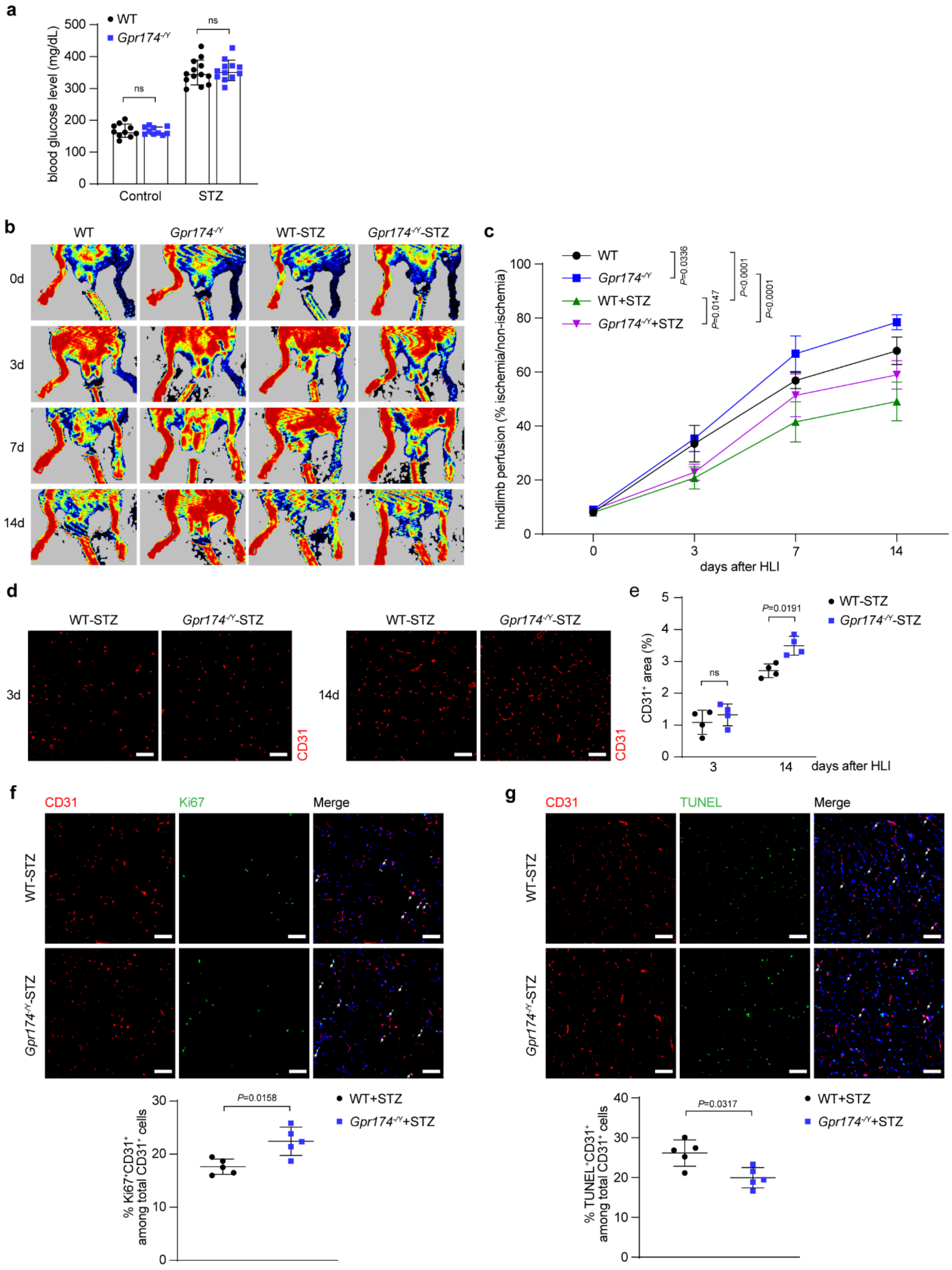

Supplementary Fig. 13. GPR174 knockout enhances blood flow recovery after HLI in diabetic mice. a blood glucose levels in STZ-induced diabetic mice (n=10 for WT mice; n=10

for *Gpr174*$^{-/Y}$ mice; n=13 for WT-STZ mice; n=12 for *Gpr174*$^{-/Y}$-STZ mice). **b**, **c** Representative laser Doppler images and quantification of hindlimb blood perfusion in WT and *Gpr174*$^{-/Y}$ diabetic mice at indicated times after HLI (n=5 for WT mice; n=5 for *Gpr174*$^{-/Y}$ mice; n=8 for WT-STZ mice; n=7 for *Gpr174*$^{-/Y}$-STZ mice). **d**, **e** Representative immunofluorescent images of CD31 staining and quantification of CD31$^+$ area in WT and *Gpr174*$^{-/Y}$ diabetic mice gastrocnemius cross sections at the indicated times after HLI (n=5). Scale bar, 50 μm. **f** Representative immunofluorescent image of CD31 (red), Ki67 (green) and DAPI (blue) and quantification of CD31$^+$Ki67$^+$ cells in muscle cross sections 3 days after HLI (n=5). Scale bar, 50 μm. **g** Representative immunofluorescent images of CD31 (red), TUNEL (green), and DAPI (blue) staining and quantification of CD31$^+$TUNEL$^+$ cells in muscle cross sections 3 days after HLI (n=5). Scale bar, 50 μm. For all statistical plots, the data are presented as mean ± SD. Two-way ANOVA with Bonferroni multiple comparisons test in (**a**). Two-way repeated measures ANOVA with Sidak's multiple comparisons test in (**c**). One-way ANOVA with Bonferroni multiple comparisons test in (**e**). Two-tailed unpaired t-tests in (**f**, **g**). Source data are provided as a Source Data file.

Supplementary Tables

Supplementary Table 1. Antibodies for Flow cytometry.

Antibody	Dilution	Cat No.	Manufacturer
CD16/CD32 Monoclonal Antibody (FRC-4G8)	1:50	MFCR00-4	Thermo Fisher Scientific
Fixable Viability Stain 510	1:1000	564406	BD Biosciences
CD45-APC/Cy7	1:400	557659	BD Biosciences
CD11b-PE/Cy7	1:400	561098	BD Biosciences

Ly6G-FITC	1:400	561105	BD Biosciences
F4/80-BV421	1:400	565411	BD Biosciences
Ly6C-APC	1:400	560595	BD Biosciences
CD3e-BV421	1:400	564008	BD Biosciences
CD4-APC	1:400	553051	BD Biosciences
CD8-PE	1:400	553033	BD Biosciences
CD31-PE	1:400	553373	BD biosciences
KLRG1-APC/Cy7	1:200	138425	Biolegend
CD90.2-PerCP/Cyanine5.5	1:200	105337	Biolegend
CD45-PE	1:200	12-0451-81	eBioscience
Foxp3-FITC	1:200	126406	Biolegend
CD45R-FITC	1:400	553088	BD biosciences

Supplementary Table 2. Antibodies for Immunofluorescence staining.

Antibody	Dilution	Sources of species	Cat No.	Manufacturer
Anti-GPR174	1:100	Rabbit	SAB4503375	Sigma
Anti-AREG	1:50	Rabbit	PA5-109404	Invitrogen
Anti-EGR1	1:50	Rabbit	4154s	CST
Anti-GFP	1:250	Chicken	ab13970	Abcam
Anti-αSMA	1:500	Mouse	A5228	Sigma
Anti-CD31/PECAM-1	1:50	Goat	AF3628	R&D Systems
Anti-Ki67	1:100	Rabbit	9129	CST
Anti-Foxp3	1:50	Rat	41-5773-82	eBioscience
Anti-VE-cadherin	1:50	Mouse	Sc-9989	Santa Cruz

Supplementary Table 3. Antibodies for immunoblot analysis.

Antibody	Dilution	Sources of species	Cat No.	Manufacturer

Anti-GPR174	1:1000	Rabbit	SAB4503375	Sigma
Anti-AREG	1:1000	Rabbit	PA5-109404	Invitrogen
Anti-EGR1	1:1000	Rabbit	4154s	CST
Anti-Bax	1:1000	Rabbit	2772s	CST
Anti-Bcl-2	1:1000	Rabbit	2876	CST
Anti-C-Caspase-3	1:500	Rabbit	19677-1-AP	Proteintech
Anti-VE-cadherin	1:1000	Mouse	sc-9989	Santa Cruz
β-actin	1:1000	Rabbit	8457s	CST

Supplementary Table 4. The primer sequences for real-time quantitative PCR.

**Gene**	**Species**	**Primer sequence**
Areg	Mus musculus	Forward: GGTCTTAGGCTCAGGCCATTA
		Reverse: CGCTTATGGTGGAAACCTCTC
Egr1	Mus musculus	Forward: TTACCCGCCATATCCGCATC
		Reverse: TGCCTCTTGCGTTCATCACT
Il-1α	Mus musculus	Forward: CGAAGACTACAGTTCTGCCATT
		Reverse: GACGTTTCAGAGGTTCTCAGAG
Il-1β	Mus musculus	Forward: AGCTCTCCACCTCAATGGAC
		Reverse: GACAGGCTTGTGCTCTGCTT
Il-6	Mus musculus	Forward: TCCATCCAGTTGCCTTCTTG
		Reverse: GGTCTGTTGGGAGTGGTATC
Il-12a	Mus musculus	Forward: TGCCTTGGTAGCATCTATGAGG
		Reverse: CGCAGAGTCTCGCCATTATGAT
Il-23a	Mus musculus	Forward: AATAATGTGCCCCGTATCCAGT
		Reverse: GCTCCCCTTTGAAGATGTCAG
Tnf-α	Mus musculus	Forward: ACGGCATGGATCTCAAAGAC
		Reverse: CGGACTCCGCAAAGTCTAAG
Cxcl1	Mus musculus	Forward: ACTGCACCCAAACCGAAGTC
		Reverse: TGGGGACACCTTTTAGCATCTT

Cxcl2	Mus musculus	Forward: TTTGCCTTGACCCTGAAGCC
		Reverse: GAGGCACATCAGGTACGATCC
Cxcl10	Mus musculus	Forward: CCAAGTGCTGCCGTCATTTTC
		Reverse: GGCTCGCAGGGATGATTTCAA
Nos2	Mus musculus	Forward: ACATCGACCCGTCCACAGTAT
		Reverse: CAGAGGGGTAGGCTTGTCTC
Tlr2	Mus musculus	Forward: CACCACTGCCCGTAGATGAAG
		Reverse: AGGGTACAGTCGTCGAACTCT
Tlr4	Mus musculus	Forward: TTTGACACCCTCCATAGACTTCA
		Reverse: GAAACTGCAATCAAGAGTGCTG
Tlr9	Mus musculus	Forward: ACGGGAACTGCTACTACAAGA
		Reverse: CCCAGCTTGACAATGAGGTTAT
Mmp3	Mus musculus	Forward: TCTGGGCTATACGAGGGCAC
		Reverse: ACCCTTGAGTCAACACCTGGA
Mmp9	Mus musculus	Forward: GCAGAGGCATACTTGTACCG
		Reverse: TGATGTTATGATGGTCCCACTTG
Fas	Mus musculus	Forward: GCGGGTTCGTGAAACTGATAA
		Reverse: GCAAAATGGGCCTCCTTGATA
Tgf-β	Mus musculus	Forward: CTTCAATACGTCAGACATTCGGG
		Reverse: GTAACGCCAGGAATTGTTGCTA
Vegfα	Mus musculus	Forward: GCACATAGAGAGAATGAGCTTCC
		Reverse: CTCCGCTCTGAACAAGGCT
Arg1	Mus musculus	Forward: CTCCAAGCCAAAGTCCTTAGAG
		Reverse: AGGAGCTGTCATTAGGGACATC
Mgl1	Mus musculus	Forward: CAATGTGGTTAGTTGGATCGGC
		Reverse: CCCAGTTCTTAAAGCCTTTCTCA
Mgl2	Mus musculus	Forward: TTAGCCAATGTGCTTAGCTGG
		Reverse: GGCCTCCAATTCTTGAAACCT
Il-10	Mus musculus	Forward: AGCCTTATCGGAAATGATCCAGT

		Reverse: GGCCTTGTAGACACCTTGGT
Cxcr4	Mus musculus	Forward: TGGAACCGATCAGTGTGAGT
		Reverse: GGGCAGGAAGATCCTATTGA
Pdgfb	Mus musculus	Forward: AGCAGAGCCTGCTGTAATCG
		Reverse: GGCTTCTTTCGCACAATCTC
Nrp1	Mus musculus	Forward: TCCTGGGAAACTGGTATATCTATGA
		Reverse: CATTCCAGAGCAAGGATAATCTG
Mrc1	Mus musculus	Forward: CTCTGTTCAGCTATTGGACGC
		Reverse: CGGAATTTCTGGGATTCAGCTTC
Ym1	Mus musculus	Forward: CAGGTCTGGCAATTCTTCTGAA
		Reverse: GTCTTGCTCATGTGTGTAAGTGA
Cd36	Mus musculus	Forward: AGATGACGTGGCAAAGAACAG
		Reverse: CCTTGGCTAGATAACGAACTCTG
Fizz	Mus musculus	Forward: CCAATCCAGCTAACTATCCCTCC
		Reverse: CCAGTCAACGAGTAAGCACAG
Msr1	Mus musculus	Forward: TTCACTGGATGCAATCTCCAAG
		Reverse: CTGGACTTCTGCTGATACTTTGT
18s	Mus musculus	Forward: CATTCGAACGTCTGCCCTATC
		Reverse: CCTGCTGCCTTCCTTGGA

Supplementary Fig. 1a

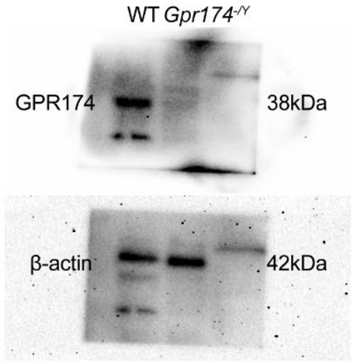

Supplementary Fig. 5d

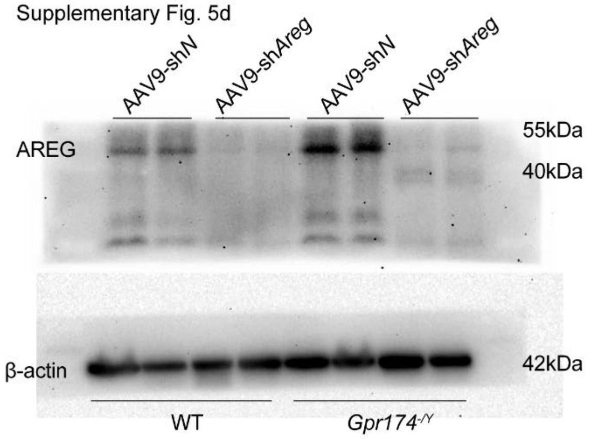

Supplementary Fig. 8c

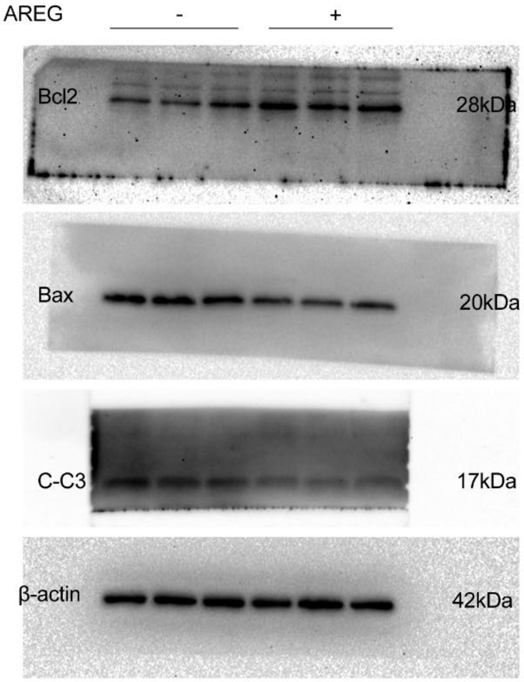

Supplementary Fig. 8g

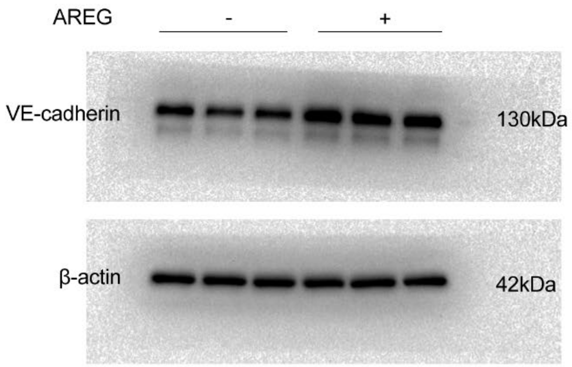

Supplementary Fig. 12d

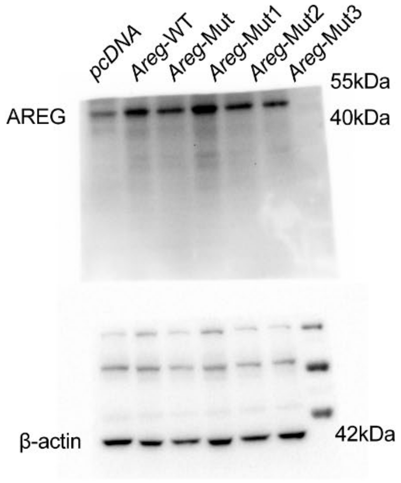

Supplementary Fig. 12c

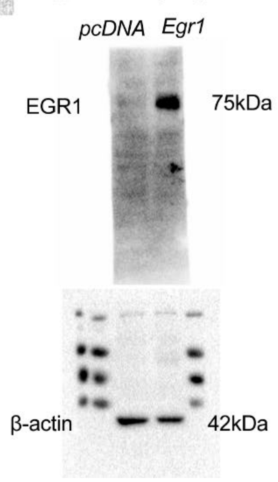

Supplementary Fig. 12b

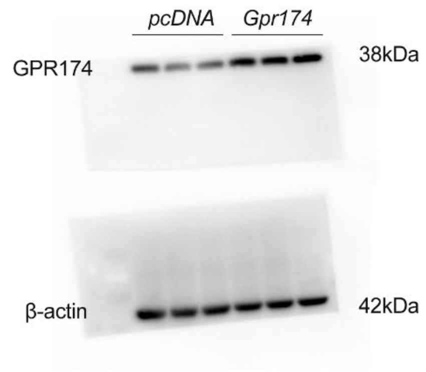

Supplement: Supplementary file 1 — Supplementary Information [file 41467_2022_35159_MOESM1_ESM.pdf]
