# Supplementary material for: GPR174 knockdown enhances blood flow recovery in hindlimb ischemia mice model by upregulating AREG expression
Source: Nat Commun. 2022 Dec 6;13:7519. doi: 10.1038/s41467-022-35159-8 (PMC9727025; doi:10.1038/s41467-022-35159-8)
Supplement: Supplementary file 3 — Description of Additional Supplementary Files [file 41467_2022_35159_MOESM3_ESM.pdf]

Title: Supplementary Data 1
Description: List of plasmid sequences used for Areg promoter luciferase activity assay.
